# Chest trauma experience over eleven-year period at al-mouassat university teaching hospital-Damascus: a retrospective review of 888 cases

**DOI:** 10.1186/1749-8090-7-35

**Published:** 2012-04-19

**Authors:** Ibrahim Al-Koudmani, Bassam Darwish, Kamal Al-Kateb, Yahia Taifour

**Affiliations:** 1 Department of thoracic surgery, Al-Mouassat University Hospital, Damascus, Syria; 2 Victoria Bridge, Al-Jumhoriah St, Mardam Building, Damascus, Syria

**Keywords:** Chest trauma, Rib fractures, Traffic accident, Blunt injury, Penetrating injury

## Abstract

**Background:**

Thoracic trauma is one of the leading causes of morbidity and mortality in developing countries. In this study, we present our 11-year experience in the management and clinical outcome of 888 chest trauma cases as a result of blunt and penetrating injuries in our university hospital in Damascus, Syria.

**Methods:**

We reviewed files of 888 consequent cases of chest trauma between January 2000 and January 2011. The mean age of our patients was 31 ± 17 years mostly males with blunt injuries. Patients were evaluated and compared according to age, gender, etiology of trauma, thoracic and extra-thoracic injuries, complications, and mortality.

**Results:**

The leading cause of the trauma was violence (41%) followed by traffic accidents (33%). Pneumothorax (51%), Hemothorax (38%), rib fractures (34%), and lung contusion (15%) were the most common types of injury. Associated injuries were documented in 36% of patients (extremities 19%, abdomen 13%, head 8%). A minority of the patients required thoracotomy (5.7%), and tube thoracostomy (56%) was sufficient to manage the majority of cases. Mean hospital LOS was 4.5 ± 4.6 days. The overall mortoality rate was 1.8%, and morbidity (n = 78, 8.7%).

**Conclusions:**

New traffic laws (including seat belt enforcement) reduced incidence and severity of chest trauma in Syria. Violence was the most common cause of chest trauma rather than road traffic accidents in this series, this necessitates epidemiologic or multi-institutional studies to know to which degree violence contributes to chest trauma in Syria. The number of fractured ribs can be used as simple indicator of the severity of trauma. And we believe that significant neurotrauma, traffic accidents, hemodynamic status and GCS upon arrival, ICU admission, ventilator use, and complication of therapy are predictors of dismal prognosis.

## Background

Trauma continues to be a major public health problem worldwide as it is associated with high morbidity and mortality both in developed and developing countries. Trauma also reported to be the leading cause of death, hospitalization, and long-term disabilities in the first four decades of life [1]. Thoracic trauma comprises 10-15% of all traumas [2]. Thoracic trauma directly accounts for approximately 25% of trauma related mortality and is a contributing factor in another 25% [3]. Fortunately over 80% of injuries can be managed non-operatively utilizing tube thoracostomy, appropriate analgesia and aggressive respiratory therapy [3].

Eastern Mediterranean region has one of the highest rates of trauma mortalities around the world. The World Health Organization (WHO) documented over 300,000 deaths in 2008 (9% of all world deaths) [4].

The etiological pattern of chest trauma varies worldwide with many environmental and socio-political factors. Road traffic accidents (RTAs) remain the cause of most chest trauma in non-war zones.

We present the pattern, etiology, management, and outcome of chest trauma in Damascus university hospital over 11-years period.

## Methods

Al-Mouassat university hospital is an 820 beds tertiary referral center for Damascus city and it is the teaching hospital for Damascus University. It provides adults and pediatric chest trauma care in its emergency department. We included all patients who were hospitalized due to non-iatrogenic chest injuries between January 2000 and January 2011 in our study (a total of 888 consequent cases). The hospitalization criteria were intra-thoracic injury, clinically significant rib cage injury (even one rib fracture), or clinical suspicion of significant thoracic injury like subcutaneous emphysema. We excluded from our study all patients who arrived dead or early in the emergency room, patients who did not complete their treatment in our hospital, isolated laryngeal or cervical injuries, esophageal and tracheal injuries due to foreign body swallowing or aspirating, and non-traumatic injuries to the chest (burns, electrical shocks, etc.).

There were 773 males (87%), and 115 females (13%), male to female ratio was (6.7:1). The mean age was 31 ± 17 years, ranging from few months to 95 years. Blunt and penetrating injuries were documented in (n = 513, 58%) and (n = 375, 42%) patients, respectively.

Data were collected prospectively and analyzed retrospectively. Patients' data were analyzed according to age, gender, trauma type, etiology of the trauma, thoracic and extra-thoracic injuries, hospital Length Of Stay (LOS), Intensive Care Unit (ICU) admission, mechanical ventilation, surgical interventions, morbidity, and mortality.

The injured patients were first triaged by a specialist in emergency medicine in the emergency department. Patients were then referred to the thoracic surgeon if needed. Patients with poor condition or those with flail chest were admitted to the ICU and mechanical ventilation was used for respiratory deficiency or severe neurotrauma. All patients had analgesics, and mucolytic treatment, provided with respiratory physiotherapy. Thoracotomy indications were: initial chest tube output > 1500 ml or hourly output > 200 ml for 4 hours, hemopericardium, prolonged air leakage, radiologic or endoscopic indicators of injury in esophagus, trachea and bronchi, heart and great vessels.

This is a descriptive study. Statistical calculations were performed using the GraphPad Prism^® ^V.5 software for Windows (GraphPad Software, Inc, La Jolla, CA, USA). All values were expressed as mean ± S.D. (P) value of less than (0.05) was considered significant.

Ethical approval was obtained and written informed consent was obtained from the patients for publication of this report and any accompanying images.

## Results

**Figure 1:**
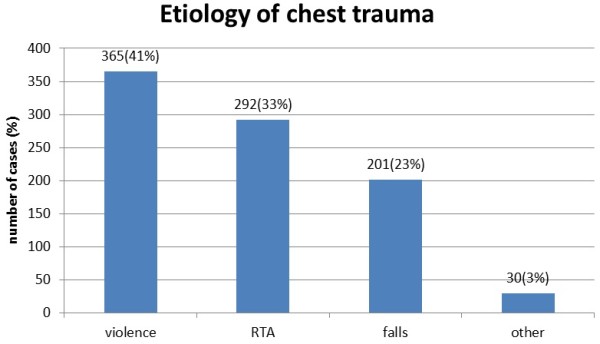
**The etiology of chest trauma**.

The main causes of chest trauma were violence (n = 365, 41%), RTAs (n = 292, 33%), and falls (n = 201, 23%). other causes (n = 30, 3%) included: work, domestic, and sport accidents Figure 1.

Road traffic accidents occurred in (n = 292, 33%) patients, 40% of which were pedestrians. In July 2004 a new traffic laws was enforced Syria restricting the speed limits and enforcing seat belt application which reduced the incidence of traffic accidents in our institution from mean of 40 to 26 patient per year before and after 2004 respectively (P = 0.02), and also reduced the mean hospital LOS (P = 0.0002), and the number of fractured ribs (P < 0.0001).

**Figure 2:**
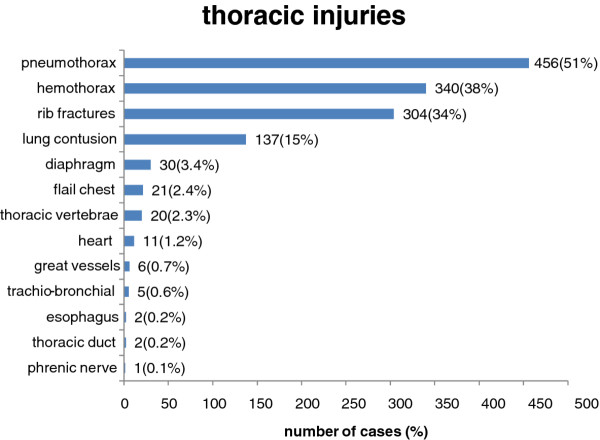
**Types of thoracic injury**.

Pneumothorax, hemothorax, rib fractures, and pulmonary contusion were the most frequent injuries accounting for 51%, 38%, 34%, 15%, respectively. Other injuries were relatively rare; diaphragmatic injury was diagnosed in (n = 30, 3.4%), flail chest in (n = 21, 2.4%), penetrating heart injury in (n = 11, 1.2%), trachio-bronchial injury in (n = 5, 0.56%), esophageal injury in (n = 2, 0.2%), thoracic duct injury in (n = 2, 0.2%) Figure 2.

Rib fractures presented in (n = 304, 34%) in this series. 75% of which had 4 or less rib fractures. 31% of rib fracture cases were isolated, whereas 69% had another intra-thoracic injury (hemo/Pneumothorax, lung contusion) which increased as the number of fractured ribs increased: (60%, 71%, 79%) for patients who had (1-2, 3-5, > 5) fractured ribs, respectively (P = 0.02). 43% of rib fractures cases were associated with extra-thoracic injuries (mostly extremity and head injuries), which also increased as the number of fractured ribs increased. The mean hospital LOS increased as the number of fractured ribs increased (4, 5, 7 days) for patients who had (1-2, 3-5, > 5 ribs), respectively (P = 0.0002). The mortality rate was proportional to the number of fractured ribs as well (P = 0.035). Delayed presentation of pneumo\hemothorax and lung contusion accompanied 5% of rib fractures cases which had no relation to the number of fractures. 18% of lower ribs fractures (9-12) were associated with intra-abdominal injuries (liver, spleen, kidney), whereas 6% of first rib fracture cases were associated with vascular injuries. 26 of rib fracture cases occurred in children; only 2 of which were non-complicated, while pneumothorax associated 20 cases, hemothorax 12 cases, lung contusion 10 cases. Extra-thoracic injuries associated 15 cases.

Penetrating heart injuries occurred in (n = 11, 1.2%) patients between 10 and 40 years of age, 10 of which were due to violence accidents (knife stabbing in 8 cases and gun shots in 2 cases), and primary surgical repair with pericardial or Teflon pledgets were done without cardio-pulmonary bypass. Atrial injury was documented in one case, but most of the injuries were ventricular injuries and in one case the injury was through-and-through the right ventricle. 9 patients arrived to the hospital with profound shock, and most of them were resuscitated successfully including one case of short term traumatic arrest, but unfortunately 2 patients died (mortality rate: 18%).

**Figure 3:**
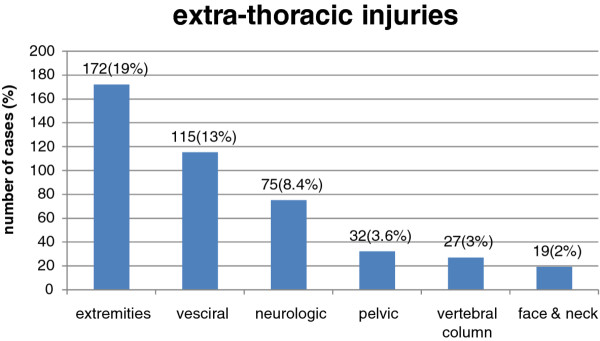
**Associated extra-thoracic injuries**.

Extra-thoracic injuries occurred in (n = 317, 35%) patients; extremities in (n = 172, 19%), visceral in (n = 115, 13%), neurotrauma in (n = 75, 8%). The mean hospital LOS was longer (5.4 ± 4.6 days) comparing to pure thoracic injury group (4.2 ± 4.5 days) (P = 0.0004) Figure 3.

Tube thoracostomy was performed in (n = 496, 56%), thoracotomy in (n = 51, 5.7%), Video-Assisted Thoracic Surgery (VATS) in (n = 6, 0.7%). Extra-thoracic intervention in (n = 164, 18%); mostly laporatomy in (n = 111, 12.5%). The main indication for surgical exploration was uncontrolled hemorrhage, re-exploration was performed for 2 patients Table 1.

**Table 1:** Thoracic injuries discovered by surgical exploration

	n	Blunt	Penetrating	*P*
**Cardiac injury**	11	0	11	< 0.0001

**Intercostals vessel injury**	13	1	12	0.0002

**Pulmonary laceration**	19	3	16	0.0002

**Diaphragm rupture**	31	10	21	0.005

**Internal mammary vessels**	4	0	4	0.03

**Chest wall injury**	13	3	10	0.02

**Fibrinous Hemothorax**	13	7	6	NS

**Pulmonary helium injury**	3	3	0	NS

**Subclavian vessels injury**	3	1	2	NS

**Bronchial rupture**	3	3	0	NS

**Tracheal laceration**	2	0	2	NS

**chylothorax**	2	1	1	NS

**Esophageal rupture**	1	0	1	NS

The mean hospital LOS was 4.5 ± 4.6 days, ranging from 1-45 days. Blunt trauma victims stayed in hospital for longer time (5.2 ± 0.2 days) than penetrating trauma patients (3.9 ± 0.2 days) (P < 0.0001). The mean hospital LOS was more when complication of treatment occurred (13.5 + 8.7 days) (P < 0.0001).

Intensive care unit admission was required for (n = 77, 8.6%) patients. Mechanical ventilation was required for (n = 43, 4.8%) for a mean time of 7.2 ± 6 days.

Treatment complications occurred in (n = 78, 8.7%) patients. The most common complications were prolonged air leak (39 cases), followed by clotted hemothorax (15 cases) Table 2.

**Table 2:** Complications of therapy

Morbidity	Number of cases	Percentage (%)
**Prolonged air leak**	39	4.4

**Clotted Hemothorax**	15	1.7

**Atelectasis**	7	0.8

**Pneumonia**	5	0.56

**Empyema**	5	0.56

**ARDS**	3	0.33

**Surgical bleeding**	2	0.22

**Peptic ulcer**	2	0.22

Mortality occurred in (n = 16, 1.8%). 56% of which occurred early (within 48 hours), and 44% occurred late in the course of trauma. 56% of mortality cases arrived to the hospital with profound shock. Glasgow Coma Scale (GCS) upon arrival was (14.5 ± 0.06) for survivals and (9.4 ± 1.2) for non-survivals (P < 0.0001). We found that mortality rate was higher in traffic accidents comparing to other causes of trauma (P = 0.045), when neurotrauma associated thoracic injury (P = 0.044), when ICU admission was required (P < 0.0001), when ventilator was required (P < 0.0001) and when a complication of therapy occurred (P = 0.044).

Out of 888 patients in this study there were 105 pediatric patients (under 14 years of life), blunt trauma accounted for 70% of them. 40% of pediatric trauma caused by falls whereas RTAs caused 36%, other causes included landmines (8%) which were residual in Al-Qunaitira region in south west Syria, domestic accidents, assaults and suicide attempts.

58% of thoracic injuries in children had pneumothorax, 37% had hemothorax, 32% had lung contusion, and 25% had rib fracture(s). Extra-thoracic injuries were present in 50% of children chest trauma cases (abdomen 24%, head 16%, extremities 9%). 6 children underwent thoracotomy, and 3 of them had laparotomy. Surgical findings were; diaphragmatic injury (5 cases), heart injury (1 case), bronchial injury (1 case), lung parenchymal injury (3 cases one of them underwent lobectomy). Tube thoracostomy were performed for 56 children, 2 of which were bilateral. There was one pediatric mortality case.

## Discussion

Trauma has the tendency to affect young males in the productive period of life [5][6][7]. Many series derived from the Middle East region had even younger patients [8][9][10][11] comparing to studies done in the developed countries [12][13][14]. Blunt trauma was more frequent than penetrating trauma in our series which is compatible with other series [2][5][11][12][15]. The literature suggest that traffic accidents is the leading cause of chest trauma in most series [2][5][8][9][10][11][12][13][14][16]. But violence was the leading cause in our series and exceeded traffic accidents. The current study is an institutional report, and this result can not be generalized until epidemiological or multi-institutional study is performed. Also note that this study was performed before the latest unrest in Syria.

Seat belt is the most effective method for reducing injuries due to traffic accidents [17], we experienced that seat belt enforcement (among other traffic laws) has reduced the severity of trauma among traffic accident victims.

Rib fractures occurred in 34% of patients and was the most common type of injury due to blunt trauma in our series which was comparable with other series [5][8][12][16]. It is widely accepted that the number of fractured ribs indicates the severity of trauma and closely correlates with morbidity and mortality [7][18][19][20]. Many studies could prove this correlation for mortality and some indicators of morbidity. Our results were significant also for mortality, hospital LOS, thoracic, and extra-thoracic injuries. Flagel et al. reported the increase of mortality, pneumonia, Acute Respiratory Distress Syndrome (ARDS), pneumothorax, empyema, ICU LOS, and hospital LOS for each additional rib fracture on over 730,000 patients [18]. Children's rib fractures are less common than adults because they have more elasticity of their bones than adults, however when rib fracture occurs it indicates severe trauma in children and associates with high rates of thoracic and extra thoracic injuries.

Follow up is essential for all patients who had the diagnosis of rib fractures. Most rib fractures cases (69%) are associated with other thoracic injury mainly hemo\pneumothorax and lung contusion, 5% of which diagnosed after 24 hours of trauma. This rate showed no significant difference according to the number of fractures, which dictates follow up for 2-3 days for all rib fracture cases even with one rib fractured and no other thoracic injury at the moment of admission. Follow-up could be as out-patient basis according to most authors, however some authors recommend hospitalizating of all patients [19].

Flail chest was diagnosed in (n = 21, 2.4%) all of them were adults. While the indications and value of surgical fixation is still debated [20], we performed surgical fixation in two cases in the course of thoracotomy for another indication.

We had 30 (3.4%) patients diagnosed with diaphragmatic injury, 33% of which due to blunt injury and were mainly front seat passengers RTAs and left sided injury, and 67% of diaphragmatic injuries due to thoracoabdominal wounds where the associated injuries dictates the surgical approach for repair (in our series most diaphragmatic injuries were repaired through laparotomy). It is not rare to miss a diaphragm injury since non-invasive diagnostic measures have little value to diagnose non-complicated diaphragmatic injury, while late diagnosis has significant morbidity and mortality. One third of thoracoabdominal wounds associated with diaphragmatic injuries [21][22]. VATS is an accurate method for identifying diaphragmatic injuries and can be used when there is no indication for laparotomy or thoracotomy, but its indications is still unclear, Freeman et al. identified five independent predictors of diaphragmatic injuries after penetrating chest trauma which can be used as indications of VATS; abnormal chest radiograph, associated intra-abdominal injuries, high-velocity mechanism of injury, entrance wound inferior to the nipple line or scapula, and right-sided entrance wound [22].

Trachio-bronchial injuries reported in 1-3% of trauma victims in the literature, most of these cases die before arriving at a hospital [23]. Trachio-bronchial injuries were documented in 5 (0.56%) patients in our series, cervical trachea injuries (2 cases) were due to penetrating trauma, whereas bronchial injuries (3 cases) were due to high velocity blunt trauma, right main stem injury was identified in one case after one year of high velocity multiple trauma. The surgical repair was successful in all cases, and no complication or mortality were documented. Kiser et al. reviewed all patients with blunt tracheobronchial injuries published in the literature and he stated that delayed treatment does not affect successful surgical repair [23].

Most victims of heart injuries die at the scene of accident, even patients who arrive at hospital alive have high mortality rate (10-50%) [24], our results on penetrating cardiac injuries were comparable to other series where most cases are caused by violence using sharp weapons or less frequently firearms, the victims are mostly males under 40 years, most injuries occurred in one chamber more frequently in right ventricle, primary repair can be used in most cases without cardiopulmonary bypass, mortality rate depends mainly on the nature of the injury and the hemodynamic status upon arrival [24][25].

Tube thoracostomy was the main treatment modality for the majority (56%) of patients, open thoracotomy was performed in 5.7% of patients, these ratios are similar to previously published results [2][6][7][8][10][11][15][16]. Intrathoracic hemorrhage was the main indication of early thoracotomy, while late thoracotomies were performed to evacuate clotted hemothorax, or to repair diaphragmatic injuries in most cases.

The relatively low mortality rate (1.8%) in this study may be interpreted by high pre-hospital mortality rate, other studies are needed to estimate the real mortality rate after chest trauma in Syria. We did not include pre-hospital or early hospital mortalities because of lack of data, also we did not analyze pre-hospital transportation times.

The predictors of mortality were; RTAs, hemodynamic status upon arrival, GCS upon arrival, neurotrauma, ICU admission, ventilator use, and complication of therapy. The predictors of prolonged hospital LOS were; blunt trauma, number of fractured ribs, associated extra-thoracic injuries, and complications of therapy.

## Conclusions

Chest trauma is a major health problem (especially among young males) since it has high morbidity and mortality rate (about 10% in this series).

Road safety and strict traffic laws (especially seat belt enforcement) contribute to the reduction of the incidence of chest trauma caused by RTAs and the reduction of the severity of injuries and mortality following RTAs when happened.

Civil violence is a main cause of chest trauma, and was the most common cause of chest trauma in this series rather than RTAs, more studies need to be performed to know the real ratio of violence in normal civil situations in Syria.

Among several trauma severity score systems; simple fractured ribs number is a precise and fast one which indicates the severity of thoracic and extra-thoracic injuries and predicts the prognosis.

According to our analysis; mortality predictors were: RTAs, hemodynamic status upon arrival, GCS upon arrival, neurotrauma, ICU admission, ventilator use, and complication of therapy. The predictors of prolonged hospital LOS were: blunt trauma, number of fractured ribs, associated extra-thoracic injuries, and complications of therapy.

## Abbreviations

LOS: Length of stay; GCS: Glasgow coma scale; ICU: Intensive care unit; RTA(s): Road Traffic accident(s); ARDS: Acute respiratory distress syndrome; VATS: Video-assisted thoracic surgery.

## Competing interests

The authors declare that they have no competing interests.

## Authors' contributions

IAK contributed in study design and data analysis and wrote the draft of the manuscript and performed the literature review. BD supervised the study and contributed in study design, data analysis, manuscript writing & editing. KAK and YT contributed in study design, data analysis, manuscript writing & editing. All the authors read and approved the final manuscript.
